# Vulnerability of Parkinson's Patients to COVID-19 and Its Consequences and Effects on Them: A Systematic Review

**DOI:** 10.1155/2023/6272982

**Published:** 2023-04-24

**Authors:** Sorayya Rezayi, Meysam Rahmani Katigari, Leila Shahmoradi, Mehrbakhsh Nilashi

**Affiliations:** ^1^Health Information Management and Medical Informatics Department, School of Allied Medical Sciences, Tehran University of Medical Sciences, Tehran, Iran; ^2^Health Information Technology Department, Saveh University of Medical Sciences, Saveh, Iran; ^3^UCSI Graduate Business School, UCSI University, No. 1 Jalan Menara Gading, UCSI Heights, 56000 Cheras, Kuala Lumpur, Malaysia

## Abstract

**Introduction:**

Parkinson's disease (PD) is the second most common neurological disorder. Patients with PD were affected by the COVID-19 pandemic in many different ways. This study's principal purpose is to assess PD patients' vulnerability to COVID-19 and its consequences.

**Method:**

This systematic review was performed based on Preferred Reporting Items for Systematic Reviews and Meta-Analyzes (PRISMA) guidelines. A thorough search was conducted in the Medline (through PubMed) and Scopus databases from inception to January 30, 2022. The Joanna Briggs Institute (JBI) critical appraisal checklist was used to evaluate the studies.

**Results:**

Most of the studies (38%) had been conducted in Italy. Of the total number of studies, 17 (58%) were cross-sectional, seven (22%) were cohort, four (12%) were quasiexperimental, two (6%) were case-control, and one (3%) was a qualitative study. The PD duration in patients ranged from 3.26 to 13.40 years (IQR1: 5.7 yrs., median: 3.688 yrs., and IQR3: 8.815 yrs.). Meanwhile, the sample size ranged from 12 to 30872 participants (IQR1: 46, median: 96, and IQR3: 211). Despite worsening PD symptoms in the targeted population (persons with COVID-19 and Parkinson's disease), some studies found PD to be a risk factor for more severe COVID-19 disease. There are many adverse effects during the pandemic period in PD patients such as abnormalities of motor, nonmotor functioning, clinical outcomes, activities of daily living, and other outcomes.

**Conclusion:**

This study confirmed the negative effect of the COVID-19 pandemic on health-related quality of life and its determinants in patients with PD and their caregivers. Thus, due to the worsening symptoms of PD patients in the current pandemic, these people should be given more care and supervision to minimize their coronavirus exposure.

## 1. Introduction

Parkinson's disease (PD) is the second most common neurological disorder. Patients with PD were affected by the COVID-19 pandemic in many different ways. Although the exact cause of PD is unknown, many researchers believe that factors such as genetics and environment lead to the gradual degeneration of neurons in sensitive areas of the brain and the development of PD [1]. PD affects 1 to 2 out of every 1,000 people in the population. Its prevalence increases with age and affects 1% of the population over the age of 60 [2]. From 1990 to 2019, the global and regional burden of Parkinson's disease increased. PD has had the most rapid growth in incidence and disability among neurological disorders in recent years, becoming one of the most significant causes of disability worldwide [3].

The continuation of the COVID-19 pandemic has raised numerous concerns about the increasing vulnerability of people with chronic diseases, especially neurological diseases [4]. COVID-19 can affect patients with a pre-existing neurological disorder, whether acquired or inherited. This effect is one of the significant issues facing neurologists in recent months. Also, concerns have been raised in long-term care centers about the vulnerability of PD patients to COVID-19. This is also true for most neurological diseases, which means that patients suffering from a debilitating neurological disorder are at greater risk of other diseases, especially COVID-19. But there is still not enough data to confirm this claim [5].

As a risk factor, PD can negatively affect the prognosis in cases of COVID-19. COVID-19 causes pharmacodynamic changes, such as the interaction between dopaminergic and renin-angiotensin systems in the striatum and systemic inflammation responses in PD patients, leading to the worsening of PD and exacerbation of symptoms. Also, factors such as disease-related weakness and aging may make these patients weaker than others in society. Antonini and colleagues stated that patients with PD would have a higher mortality rate if they developed COVID-19 [6]. Stress, anxiety, depression, and social isolation can also have devastating effects on PD symptoms, leading to the worsening of the disease [7]. Symptoms of the exacerbated disease include impaired motor function, an increased need for daily doses of levodopa, fatigue, cognitive impairment, and sleep disturbances. Nevertheless, the evidence for the effect of COVID-19 outcomes in patients with PD is not yet conclusive, and currently, PD and additional motion disorders have not been recognized as risk factors for COVID-19 [7].

Given that PD is a common neurological disorder in developed and developing societies, preventing the exacerbation of symptoms and declining quality of life is one of the most critical actions that policymakers should consider. In the current context of the COVID-19 pandemic worldwide, some studies have suggested that the physical and cognitive symptoms of PD patients have worsened because the current pandemic has prevented them from continuing their rehabilitation programs as usual [8–11]. Some studies have also suggested that COVID-19 disease has a more devastating effect on PD patients, leading to the worsening of vital signs compared to other patients [4, 12–14]. All these statements have been stated in recent studies, but for a more detailed review and evaluation of its various dimensions, a comprehensive and more detailed review is needed. So, it needs to be clarified precisely whether the COVID-19 pandemic has affected PD patients or not. By knowing the extent of COVID-19's impact and consequences, we can think of appropriate solutions to prevent such effects, reduce harm, and ultimately improve patients' health. Therefore, there is a need for a comprehensive systematic review to investigate the impact of the COVID-19 pandemic on PD patients. This systematic review endeavors to answer the following questions: RQ1: what are the symptoms and consequences of PD that may be exacerbated by COVID-19? RQ2: what are the effects and consequences of the COVID-19 pandemic for PD patients?

### 1.1. Research Problems and Objectives

According to our overview, several studies have examined the effects of the COVID-19 pandemic on PD patients. In most cases, studies have shown that the COVID-19 pandemic either directly through the individual's illness or indirectly through lifestyle changes and quarantine problems has caused various problems for PD patients. Nevertheless, the effects of COVID-19 on PD patients have not yet been definitively examined in any comprehensive study. The COVID-19 pandemic has disrupted PD-related care, including medical care, exercise, and social activities. Some medical centers, even Parkinson's clinics, have closed their services due to the pandemic. Impaired medical care can lead to irregular hospital visits and more problems with PD medications because they require a prescription, which may force PD patients to reduce or discontinue their medications. It can also worsen the symptoms of PD, which increases the mortality rate of PD patients. It also has more devastating effects on their condition if they are infected with COVID-19. The principal purpose of this study is to assess the vulnerability of PD patients to the COVID-19 pandemic and its consequences. The purpose of this study is to (1) consider the extent of change in signs and symptoms of PD due to COVID-19 (direct impact of COVID-19); (2) examine the various consequences of COVID-19 lockdown on patients with PD (the indirect effect of COVID-19).

## 2. Methods

The current study is a systematic review. The study protocol was written together with the research team. In this protocol, the study method was designed in six steps. The first step was to determine the inclusion and exclusion criteria. The second step was designing the search strategy. The third step was to select the studies that fit the purpose of the research, and the next step was to collect and extract the relevant data from the included studies. The assessment of the studies and the synthesis of the results were the two final steps, which are all explained in detail in the following. Also, this systematic review was performed based on the Preferred Reporting Items for Systematic Reviews and Meta-Analyzes (PRISMA) proposed by Zhang et al. [15].

### 2.1. Eligibility Criteria

#### 2.1.1. Inclusion Criteria

PICO is a useful tool for conducting qualitative reviews and asking focused clinical questions. A reliable and comprehensive query should have four components that recognize the patient problem or population (P), the intervention (I), the comparison (C), and the outcome (O). According to the PICO tool, the inclusion criteria in this systematic review include the following:

- Population: the study population in this systematic review comprised patients with PD OR COVID-19 patients with PD as a comorbidity
- Intervention: a group of COVID-19 patients with PD as their comorbidity OR PD patients
- Comparison: a group of patients without PD
- Outcome: in-hospital consequences included morbidity or severe COVID-19 infection OR self-reported changes in symptoms in patients without COVID-19

#### 2.1.2. Exclusion Criteria

1. Studies published in a non-English language
2. No journal articles and proceedings such as review papers, letters, and book chapters
3. Articles that lack full abstracts for review
4. Investigations in which the effects, evaluation, and approaches (clinical assessment or self-report) have not been reported or have not been satisfactorily clarified

### 2.2. Information Sources and Search Strategy

A systematic and comprehensive search was conducted in two scientific databases: Medline (through PubMed) and Scopus, to determine relevant papers published from inception to January 30, 2022, with terms of Medical Subject Headings (MeSH) related to COVID-19 and PD.  Table 1 illustrates the applied terms or keywords used in the search strategy and the coverage dates for each database. We utilized reference manager software (EndNote X8, Thomson Reuters) to organize references and eliminate duplicates.

### 2.3. Study Selection

**Figure 1:**
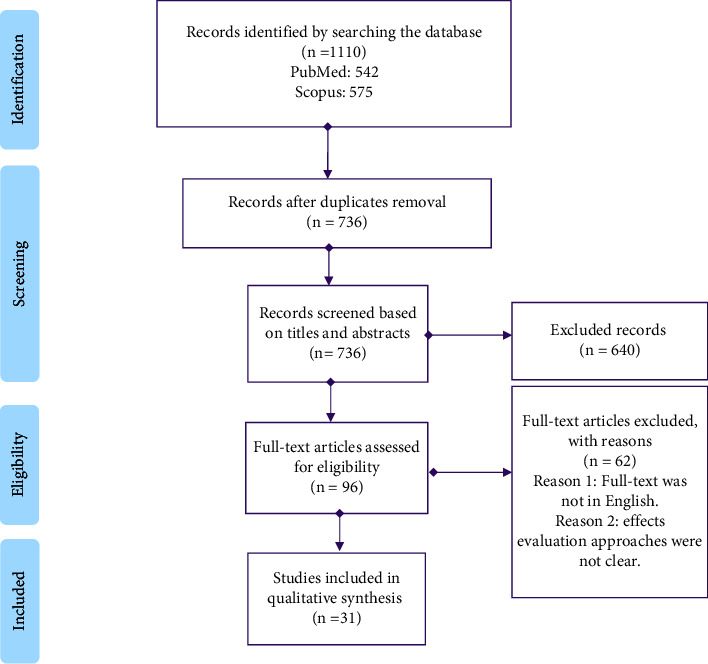
The flow diagram of determining, selecting, and screening papers based on PRISMA.

The selection process was performed in two stages. In the first stage, two reviewers (SR and MR) independently filtered the titles and abstracts of the retrieved citations. At this stage, articles that did not meet the inclusion criteria were removed. In the second stage, the full text of the articles was retrieved and reviewed and two reviewers confirmed their relevance. In the event of disagreement, the third and fourth reviewers (LS and MN) were consulted to solve the problem through a consensus method. In Figure 1, the screening process is depicted by the 2020 PRISMA checklist.

### 2.4. Data Collection Process and Data Items

**Figure 2:**
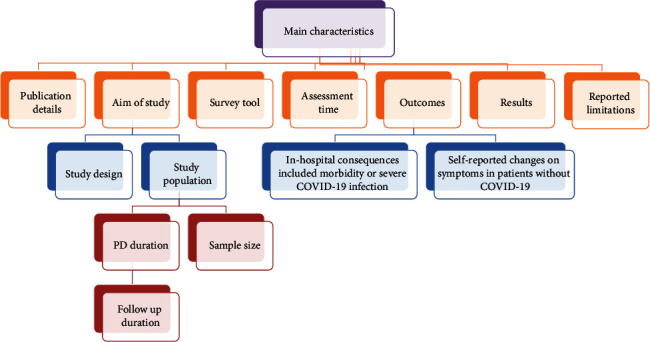
Main characteristics of the included papers.

Two reviewers (SR and MR) collected the required information from the chosen examinations. The accuracy of the accumulated information was verified by a reviewer (LS). In the last phase, a third reviewer (MN) assessed and solved any conflicts. The main features of the included papers that were mined and synthesized were entered into a structured form in Excel. In Figure 2, the characteristics of chosen articles are depicted.

### 2.5. Study Risk of Bias Assessment

The Joanna Briggs Institute (JBI) critical appraisal checklist can be used for assessing all types of studies' methodological conduct or reporting. This tool has eight questions, which can be answered with four choices: 1: yes; 2: no; 3: unclear; and 4: not applicable. Each “yes” answer obtains one score, and if 70% of the questions are responded with “yes” in research, the risk of bias is considered “low.” Also, if 50% to 69% of them are answered yes, the risk of bias is “moderate,” and finally, if less than 50% of the questions responded “yes,” the risk of bias is considered as “high” [16]. Two authors (SR and MR) completed this checklist, and in the event of a controversy between the two authors, the disagreement was resolved via discussion with the third and fourth referees (LS and MN).

### 2.6. Synthesis of Results

Meta-analysis was not used in this systematic review, as the methods and methodology of reporting results in chosen papers were heterogeneous.

## 3. Results

### 3.1. Results of the Search

A total of 1117 articles were found in the initial search, and after removing duplicates, 736 papers were left. Authors mined the title, abstract, and keywords of selected articles, so 96 articles were identified for further review. After viewing the full text of these articles and focusing on our objectives, 31 papers were included in this review. Given that the purpose of this systematic review was to examine the vulnerability of PD patients to COVID-19, the key results and characteristics are presented in three Tables (Tables 2–4). In Table 2, the study group consisted of PD patients who suffered from various injuries. In Table 3, the study group is PD patients who have COVID-19, and the consequences of the disease for them have been studied. In Table 4, the target group is both PD patients (without COVID-19) and PD patients with COVID-19 in a combined form.

### 3.2. Quality Assessment of the Included Papers

**Figure 3:**
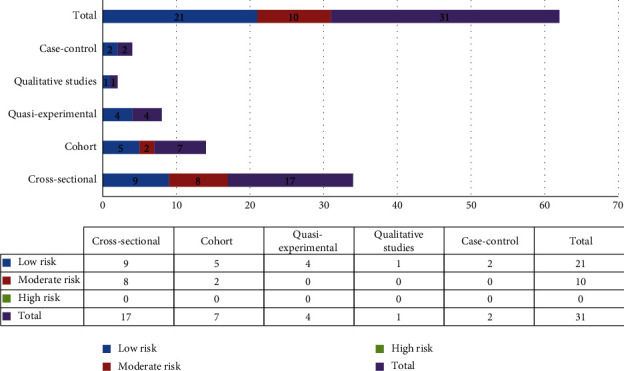
Quality assessment of the included papers.

Various studies with different designs were entered into this systematic review, and they were evaluated with an appropriate scale. The Joanna Briggs Institute Critical Appraisal (JBI) checklists were used for assessing cross-sectional, case-control, cohort, quasiexperimental, and qualitative studies. As we can see in Figure 3, twenty-one studies were considered to have a low risk of bias and ten were considered to have a moderate risk of bias.

### 3.3. Publication Analysis

#### 3.3.1. Distribution of Papers by Published Year/Month and by Their Conducted Countries

**Figure 4:**
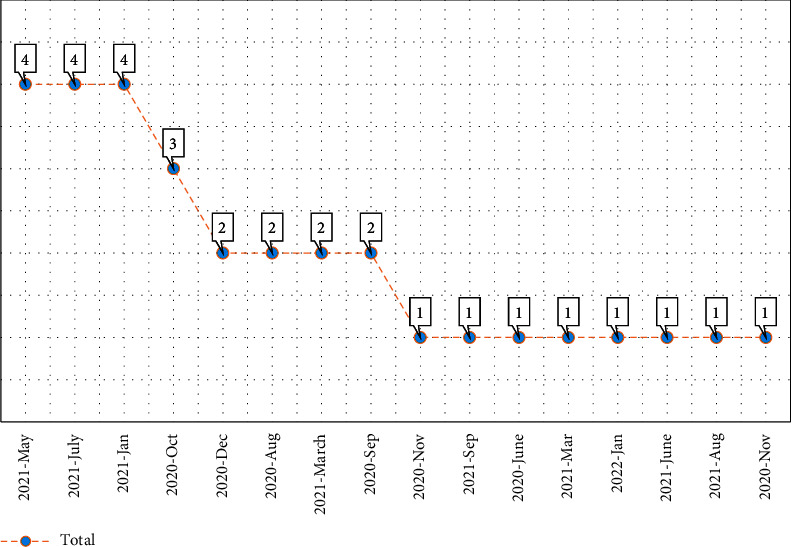
The distribution of citations by publication year/month.

**Figure 5:**
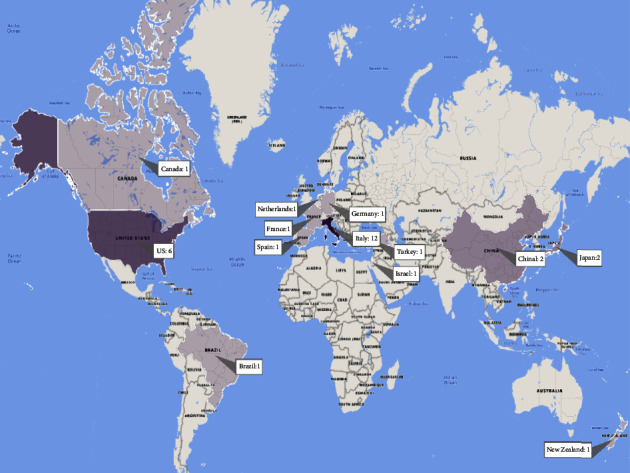
The distribution of papers based on countries.

The final distribution of papers indicates that 31 academic citations met the inclusion criteria. As can be seen in Figure 4, the largest number of studies was published in 2021. Most of the papers (38.70%) were conducted in Italy (Figure 5).

#### 3.3.2. Distribution of Academic Papers by Journals, Quartile Scores, Impact Factors (IF), and Publisher

Reviewed scientific papers (*n* = 31) were retrieved from 18 journals. The impact factor (IF) and quartile scores of the journals are presented in Table 5. It is worth noting that seven of the eighteen journals (38.88%) are ranked in the first quarter. As our analysis showed, Springer had the first rank (28.57%) among the publishers.

#### 3.3.3. General Characteristics of the Included Papers

Of the total number of studies, 17 (58%) were cross-sectional, seven (22%) were cohort, four (12%) were quasiexperimental, two (6%) were case-control, and one (3%) was qualitative. In eighteen studies (58%), the study population was PD patients who had been affected by pandemic conditions (PD patients without COVID-19). Furthermore, in eleven papers, PD patients infected with COVID-19 were the study population. In two papers, PD patients with and without COVID-19 were the study population (both study populations). In five investigations, the PD duration was not declared. The PD duration ranged from 3.26 to 13.40 years (IQR1: 5.7 yrs., median: 3.688 yrs., and IQR3: 8.815 yrs.). Meanwhile, the sample size of the studies ranged from 12 to 30872 participants (IQR1: 46, median: 96, and IQR3: 211).

#### 3.3.4. Effects of the COVID-19 Pandemic on PD Patients

A total of 31 studies were included in this systematic review. In 11 of them, the target group was PD patients with COVID-19 [14, 17–26]. In these studies, the symptoms and consequences of COVID-19 in PD patients were evaluated. In 17 studies [5, 8–10, 27–39], the target group was PD patients affected by the COVID-19 pandemic, whose symptoms had been worsened. In two studies [11, 40], the target groups were PD patients with and without COVID-19. These two were reviewed for worsening Parkinson's symptoms because of their study group. We also examined whether PD was a risk factor for more severe cases of COVID-19.

#### 3.3.5. Negative Outcomes of the Pandemic in PD Patients

Table 6 shows the effect of the COVID-19 pandemic on the symptoms of PD patients in related studies. Of the 19 studies reviewed in this section [5, 8–11, 27–40], 18 studies claimed that PD patients' symptoms were severely affected by the pandemic and only one study [40] demonstrated that there was no change in the PD patient's symptoms. The results of the studies also showed adverse effects of the pandemic on PD patients' symptoms, such as abnormalities of movement (motor functioning) and nonmotor functioning (psychiatric symptomatology). Symptoms such as tremor/shaking, gait/balance disturbances, rigidity (frigidity), fatigue, pain, distress/depression, anxiety/stress, sleep disorders (insomnia), mood disturbances, and cognitive dysfunction in patients have been severely affected by the COVID-19 pandemic, and the quality of life has been considerably reduced.

#### 3.3.6. Symptoms of PD Patients with COVID-19

Table 7 shows the symptoms and consequences of COVID-19 infection in PD patients. As can be seen in the table, many symptoms, such as motor, nonmotor functioning, clinical outcomes, activities of daily living, and other outcomes, worsened during the COVID pandemic period. The results indicated that PD was reported as a risk factor for more severe COVID-19 disease in six studies out of 13 studies [11, 14, 18, 22, 24, 26]. However, others have acknowledged that there is no link between more severe COVID-19 disease and Parkinson's disease [17, 19–21, 23, 25]. Also, the results of one study were not clear [40].

## 4. Discussion

The main goal of our systematic review was to describe and examine the vulnerability of PD patients to COVID-19 and its consequences and effects on them. To our knowledge, this study primarily focuses on identifying the symptoms and consequences of PD that may be exacerbated by COVID-19 and also the critical effects and consequences of the COVID-19 pandemic on PD patients. In total, 31 studies from the PubMed and Scopus databases were included in the review.

In some studies, the target groups were PD patients with COVID-19, among whom the acute symptoms and consequences of COVID-19 were evaluated [14, 17–26]. In some other studies, the target groups were PD patients affected by the COVID-19 pandemic, whose symptoms had been worsened [5, 8–10, 27–39]. However, in only two studies, the target groups were both PD patients with and without COVID-19 [11, 40].

Some studies have shown that COVID-19 is more common in people with Parkinson's than in people who do not have Parkinson's. They also showed that people over the age of 65 or those with severe Parkinson's disease have been affected rapidly by the new coronavirus [4, 9, 41]. Researchers also found that people with Parkinson's were more likely to be affected by high-risk illnesses if they get infected with COVID-19. People with Parkinson's disease may be at an increased risk for severe COVID-19 because of their weakness. The risk increases with age and the development of advanced Parkinson's disease. Lung function may be impaired due to Parkinson's disease and respiratory muscle weakness [5, 7]. Hence, recently published studies have shown that 31.9% of patients with pre-existing neurological disorders, including Parkinson's disease, have experienced the exacerbation of neurological symptoms during COVID-19 infection, regardless of age and duration of illness [42].

In the current pandemic situation, PD patients experience rigidity, fatigue, tremors, and a high level of pain. The subjective increase in tremor and rigidity is in line with previous studies that show these symptoms are susceptible to stress [20, 32, 34]. Increased pain may similarly result from increased anxiety but could also be caused by rigidity. Fatigue is a well-known consequence of psychological distress in PD [43–45].

Studies have shown that the COVID-19 quarantine and pandemic have altered the structure of health care and reduced patient access to the care they need, especially the trilogy treatments, and this has worsened the symptoms of PD patients [33]. Restrictions on access to hospital and outpatient services and interruptions in nonmedical treatments such as motor and cognitive rehabilitation and psychological support were significantly experienced by PD patients during the pandemic [45, 46]. The main problem for these patients was the lack of regular medical advice, which had a negative effect on their quality of life [31]. Also, the feeling of not having access to required services was much greater than the actual experience of not having access to these services [14, 28]. During quarantine, follow-up of medical services became problematic, and PD patients were reluctant to attend virtual therapy sessions [47, 48]. As it turned out, one of the biggest problems of PD patients during the prevention and control period was that they could not get regular advice from a physician [38]. Most of the PD patients decided to go to the hospital with their family members or relatives, and some others used social media software to contact their physicians [49, 50]. However, not all patients are able to use social media on their smartphones.

PD patients sometimes need to modify their medication regimen, which is often delayed during quarantine, causing concern for patients and their families [51, 52]. Changing medication routines, having difficulty accessing medications, and getting medication on time were also challenges that made PD patients anxious. Furthermore, these patients had difficulty obtaining diagnostic and therapeutic medical equipment [53–55].

A review of studies showed that the COVID-19 pandemic and quarantine created challenges in accessing a variety of health services and drugs, causing anxiety and concern in PD patients [56–58]. In general, the COVID-19 pandemic has had a negative impact on the quality of life of PD patients, caregivers, and families. Parkinson's patients need special attention from medical systems on how to receive medical services and psychosocial support [52, 59, 60].

Most studies have shown that the fear of getting infected by COVID-19, being confined to a limited space, and being banned from outdoor activities have reduced the physical activity of PD patients in addition to worsening their symptoms [33, 61]. In PD patients, experiencing fear can be associated with a greater risk of COVID-19 due to chronic comorbidity and is often attributed to anxiety [16, 37, 38]. During the pandemic, fear increases anxiety in healthy individuals and exacerbates the symptoms of individuals with previous psychiatric disorders [48, 62, 63]. Movement and lack of movement in patients also affect their physical, cognitive, and psychosocial health [56]. Some studies have suggested that a lack of physical activity is the main cause of loss in residual motor skills, especially the inability to perform high-level daily activities [13]. Decreased physical activity causes pain, headache, fatigue, worsening of walking patterns, reduced daily activities, sleep disorders, and insomnia [58]. Like the general population, the most common symptoms of COVID-19 in PD patients include fever or chills, cough, weight loss, and muscle aches. These people may have a fever with nonrespiratory symptoms, such as delirium or a distinct functional decline without any apparent physical symptoms [64, 65]. PD patients have also reported frequent motor symptoms such as bradykinesia, stiffness, and balance disorders, as well as nonmotor symptoms such as lack of motivation [66, 67].

By reducing physical activity, the time spent watching TV and using a mobile phone increase in patients with Parkinson's disease [68, 69]. Social distancing, loneliness, the inability to perform physical, recreational, family, and group activities, the elimination of therapeutic activities, changes in daily routine, and not continuing physiotherapy sessions or other training-motor training activities lead to physical problems, stress, depression, frustration, anxiety, decreased cognitive functions (attention, concentration, and memory), dissatisfaction with life, and even suicidal thoughts [5, 40]. Studies have shown that reducing physical activity, not continuing rehabilitation sessions, and aggravating the disease's motor symptoms also cause and exacerbate cognitive and psychological symptoms and affect the quality of life of patients and their families [70, 71].

### 4.1. Limitations and Strengths of This Study

This study has several strengths, including

1. Performing a comprehensive search strategy to identify a large number of studies
2. Reviewing and evaluating studies to extract data by three authors independently
3. Using a comprehensive tool (JBI) for evaluating the quality of selected studies

We have also encountered some limitations in this study. Our limitations and challenges were the difficulties of comparing studies due to the heterogeneity of the results and the exclusion of published studies because of their non-English language.

## 5. Conclusion

This systematic review examined the vulnerability of PD patients to COVID-19 and determined the consequences and effects of the COVID-19 pandemic on PD patients. By applying a systematic approach, the authors provided an exhaustive overview of the effects of COVID-19 on PD patients' symptoms. During the SARS-CoV-2 infection, patients with Parkinson's disease experienced worsening of both motor and nonmotor symptoms. These symptoms include the categories of abnormalities of motor and nonmotor functioning, clinical outcomes, activities of daily living, and other outcomes. In conclusion, this study confirms the negative impact of the COVID-19 pandemic on the quality of life and its determinants in PD patients and their caregivers. Thus, due to the worsening symptoms of PD patients in the current pandemic, these people should be given more care and supervision to minimize their exposure to coronavirus. The use of modern technologies such as telemedicine is one of the solutions that can be used for many of these problems.

## Figures and Tables

**Table 1:** Applied search strategy for databases.

PubMed	(“coronavirus”[MeSH] OR “coronavirus infections”[MeSH Terms] OR “coronavirus”[All Fields] OR “covid 2019”[All Fields] OR “SARS2”[All Fields] OR “SARS-CoV-2”[All Fields] OR “SARS-CoV-19”[All Fields] OR “severe acute respiratory syndrome coronavirus 2” [supplementary concept] OR “coronavirus infection”[All Fields] OR “severe acute respiratory pneumonia outbreak”[All Fields] OR “novel cov”[All Fields] OR “2019ncov”[All Fields] OR “sars cov2”[All Fields] OR “cov22”[All Fields] OR “ncov”[All Fields] OR “covid-19”[All Fields] OR “covid19”[All Fields] OR “coronaviridae”[All Fields] OR “corona virus”[All Fields]) AND (“Parkinson Disease”[Mesh] OR “Idiopathic Parkinson's Disease” OR “Lewy Body Parkinson's Disease” OR “Parkinson's Disease” OR “Primary Parkinsonism” OR “Paralysis Agitans”) Coverage dates: 2019–2022

Scopus	TITLE-ABS-KEY-AUTH ((coronavirus OR “middle East respiratory syndrome” OR “severe acute respiratory syndrome” OR “Porcine epidemic diarrhea virus” OR “Feline infectious peritonitis virus” OR “murine hepatitis virus” OR “Avian infectious bronchitis virus”) AND (“Parkinson disease” [mesh] OR “Idiopathic Parkinson's disease” OR “Lewy Body Parkinson's disease” OR “Parkinson's disease” OR “Primary parkinsonism” OR “Paralysis Agitans”)) Coverage dates: 2018–2022

**Table 2:** Key results of papers (study population = persons with COVID-19 and with PD).

Contribution	Year/month	Country	Aim of the study	Study design	PD duration	Sample size: Groups	Survey tool	Assessment time	Outcomes: In-hospital consequences (morbidity or severe COVID-19)	Main results	Reported limitations
Maria Buccafusca et al. [17]	2021-Jan	Italy	To describe researchers' observations on a population of patients with PD and COVID-19 admitted to the COVID-19 hospital	Cross-sectional	Mean = 9 yrs	*N* = 12 (6 M, 6 F) and AgeM = 73 yrs	(i) UPDRS	During hospitalization: 8th March to 30th May 2020	(i) Most of the PD patients had a long disease duration and multiple comorbidities even though SARS-CoV-2 manifestations were mild, and none required intensive care (ii) Despite lung conditions, most of PD patients had mild symptoms: 7 patients were clinically asymptomatic (58.3%); 3 patients had fever, cough, and myalgia (25%) and 2 patients had dyspnea (16%) that needed high-flow oxygen therapy	Slight complications of PD were seen. All patients were discharged during 30 days. No mortality occurred during hospitalization. The findings show that SARS-CoV-2 infection has a good prognosis in patients with PD	—
Joy Antonelle de Marcaida et al. [18]	2020-Sep	USA	To describe the demographic characteristics, presentation, management, and outcome of 36 patients at Movement Disorders Center with positive COVID-19, with the intent of exploring factors that may influence the clinical course in this patient population	Cross-sectional	Mean = 13.06 yrs	*N* = 36 (23 M, 13 F), AgeM = 74.5 yrs, PD = 22, Atypical parkinsonism = 7, and Other diagnosis = 7	(i) Telephone interview and medical record	During pandemic: 8th March to 6th June 2020	(i) 27 (75%) exhibited alteration in mental status (ii) 15 (42%) had abnormalities of movement (iii) In 61% and 31%, respectively, these were the presenting symptoms of the disease (iv) 67% required hospitalization (v) mortality rate 36%	These data suggest that hospitalization and mortality rates in patients with movement disorders after COVID-19 are higher than in the general population. Patients with movement disorders frequently presented with altered mental status, generalized weakness, or worsening mobility but not anosmia	(i) Retrospective data collection with little sample size
Chiara Sorbera et al. [19]	2021-May	Italy	First, to evaluate if PD patients are more susceptible than non-PD to take COVID-19 infection Second, to detect if the infection course is worse in PD-COVID-19+ patients versus non-PD	Cohort	Mean = 6.7 yrs	*N* = 18 PD = 13 (8 M, 5 F), AgeM = 68.1 yrs, non-PD = 5 (4 M, 1 F), and AgeM = 57.8 yrs	(i) Hoehn and Yahr scale	During pandemic: 15th February to 26th March 2020	(i) 50% (*n* = 5) were completely asymptomatic (ii) 40% (*n* = 4) showed mild symptoms of the infection such as intermittent fever, myalgia, pharyngitis (iii) No significative differences were found between the PD-COVID-19+ and PD-COVID-19- negative patients (iv) PD could be not considered as a risk factor for SARS-CoV-2 infection	PD was not identified as a primary risk factor for SARS-CoV-2 infection. Even if COVID-19 worsens motor and non-motor symptoms in PD's patients and increases the risk of death	(i) Few cohort and control patients' number (ii) Neuroimaging evaluation was avoided in order to reduce the risk of cross infection
Yaqian Xu et al. [20]	2021-Sep	USA	To report on people with PD who had suspected or confirmed COVID-19 to understand how COVID-19 manifested in PD patients	Cross-sectional	Mean = 7.8 yrs	*N* = 46 (29 M,17 F) and AgeM = 67.9	(i) Data collected from two study protocols: CUIMC registry cohort, PF survey cohort and (ii) survey includes question from coronavirus tracking survey, questions for neurological manifestations of COVID-19, UPDRS	During pandemic: CUIMC Registry cohort: 2nd April to 16th Sept 2020; PF survey cohort: 24th June to 9th July 2020	(i) many experienced worsening of pre-existing motor or non-motor: Bradykinesia, rigidity, balance, UPDRS-walking, UPDRS-mentation, UPDRS-motivation/initiative, UPDRS-handwriting, UPDRS-speech, UPDRS- dressing, UPDRS-turning in bed, UPDRS-thought disorder, anxiety, UPDRS-tremor, sleep- insomnia, UPDRS-hygiene, UPDRS-swallowing, UPDRS-cutting food, UPDRS-freezing, Constipation, pain, Urinary Dystonia, UPDRS-falling, UPDRS-depression,	Symptoms of COVID-19 in PD were similar to the general population. Motor symptoms (such as bradykinesia in 54%, stiffness in 49% and balance disorders in 44% of patients) and non-motor symptoms (eg, motivational problems in 58%, and mental disorders in 48% of patients) worsened during COVID-19 disease. Several motor and non-motor symptoms also appeared for the first time. A small number of participants reported improvement in PD symptoms during COVID-19 disease, including tremor, stiffness, balance disorders, handwriting, speech, shutdown time, insomnia, and anxiety	Depending on e-mail questionnaires likely biases us towards milder COVID-19 cases in patients (i) No PCR or serology test was performed to confirm the diagnosis and the patient was contacted by telephone
Sainz-Amo et al. [21]	2020-Oct	Spain	To evaluate clinical and demographic variables that may be associated with COVID-19 in patients with PD and those that may influence mortality and morbidity	Case control	Mean = 8.7 yrs	*N* = 211 PD + COVID-19 = 39(23 M, 16 F), AgeM = 75.5 yrs PD + NoCOVID-19 = 172(101 M, 71 F), and AgeM = 73.9 yrs	(i) Electronic medical record includes demographic, clinical features, advanced therapies, comorbidities, and institutionalization	During pandemic: 1st March to 31th July 2020	(i) the frequency of common comorbidities was similar between COVID-19+ and COVID-19− groups, with the exception of dementia, that was significantly more frequent in the group of cases (36% and 14%, (*p*=0.0013). Only institutionalization remained significantly associated with COVID-19 + group (*p* < 0.001)	The results showed that PD did not affect the severity of COVID-19. Epidemiological factors and fragility are also important causes of COVID-19 mortality in PD	(i) its retrospective nature and a relatively small sample size (ii) do not use any fragility scale, which was critical to diagnosing an increased risk of severe illness or death
Heng Zhai et al. [22]	2021-Jan	China	To determine the clinical manifestations, and outcomes of PD patients with severe COVID-19 and to further explore the risk factors associated with in-hospital mortality of PD patients in the early stage of the epidemic	Cohort	Mean = 8.6 yrs	*N* = 296, COVID-19 + PD = (3 M, 7 F), AgeMedian = 70 yrs, COVID-19 + NOPD = 286 (147 M, 139 F), and AgeM = 66 yrs	(i) EMR: clinical signs and symptoms, medical history, laboratory findings, treatment used, and outcomes	Admitted at hospital: 28th January to 29th February 2020	(i) the proportion of PD patients with cough and anorexia was significantly higher than that of patients without PD (65.38%, *n* = 187 and 19.23%, *n* = 55, respectively) (*p* < 0.05) (ii) No significant differences in lengths of hospital stay and duration of disease between patients with and without PD (*p* > 0.05)	Older people with a longer duration of PD and a later stage of PD are more severe if they get COVID-19. Also, the severity of COVID-19 and the presence of complications can greatly affect the prognosis of PD patients with severe COVID-19	(i) Small number of PD patients (ii) low proportion of COVID-19 patients with PD (iii) evaluation was not performed for a long time
Raminder Parihar et al. [23]	2021-May	USA	Researchers identified the risk factors that increase the risk of death in patients with Parkinson's disease who are infected by SARS-CoV-2	Cohort	Mean = 5 yrs	*N* = 87, EG = 53 (31 M, 22 F), AgeM = 78.7 yrs, CG = 34 (19 M, 19 F), and AgeM = 78.5 yrs	(i) Clinical examinations	Assessment during COVID-19	PD patients with SARS-CoV-2 infection had a higher mortality rate (35.8%) compared to PD patients without the infection (5.9%, *p*=0.028); there was a statistically significanthigher mortality rate in patients older than 70 years with COVID-19 than in 60–70 years old PD patients (*p*=0.044)	Mortality rate due to SARS-CoV-2 infection did not increase with age control in PD patients. However, some unalterable factors (advanced disease and age over 70 years) and alterable factors (reduction of PD drugs) put them at increased risk of mortality	(i) sample size of PD patients with and without COVID-19
Lynda Nwabuobi et al. [24]	2021-March	USA	Researchers aimed to determine clinical characteristics and outcomes in hospitalized PD individuals infected with COVID-19	Cohort	Mean = 6 yrs	*N* = 25, EG = 25 (19 M, 6 F), and AgeM = 82 yrs	(i) Clinical examinations	Assessment during COVID-19	The most common comorbidities were hypertension (72%) and mild cognitive impairment or dementia (48%). A total of 44% and 12% of individuals presented with altered mental status and falls, respectively. Mortality rate was 32% compared to 26% for age-matched controls (*p*=0.743)	People with PD who are hospitalized for COVID-19 infection are likely to be older, have advanced to midstate disease, and are receiving medication. Also, high blood pressure and cognitive impairment are comorbidities in these individuals. People with encephalopathy are at greater risk of death during hospitalization	(i) Larger cohorts of PD individuals with COVID-19 infection, including long-term follow up (ii) Small sample size (iii) A small percentage of individuals were misdiagnosed as Parkinson's disease
Roberto Cilia et al. [14]	2020-June	Italy	The main objective was to determine the effects of COVID-19 on motor and nonmotor symptoms in a community-based PD cohort	Cohort	Mean = 8.2 yrs	*N* = 48, EG = 12 (5 M, 7 F), AgeM = 65.5 yrs, CG = 36 (15 M, 21 F), and AgeM = 66.3 yrs	(i) Clinical examinations (ii) MDS-UPDRS (iii) NMSS (iv) CISI-PD	Baseline and End of study	Worsening of MDSUPDRS Part II and the Part IV total scores and the NMSS total score were explained by COVID-19 alone (*p*=0.008, *p*=0.034, *p*=0.008)	Regardless of the age and duration of the disease, patients with PD may experience significant worsening of motor and non-motor symptoms during mild to moderate COVID-19 disease	(i) sample size of PD patients with and without COVID-19 (ii) Larger cohorts of PD individuals with COVID-19 infection, including long-term follow up
Luca Vignatelli et al. [25]	2020-Dec	Italy	This study aimed to evaluate the risk of hospitalization for COVID-19 and death	Cohort	Not mentioned	*N* = 48, EG (Parkinson's disease) = 696 (409 M, 287 F), AgeM = 75.0 yrs EG (Parkinsonism) = 184 (105 M, 79 F), AgeM = 80.5 yrs CG = 8590 (5000 M, 3590 F), and AgeM = 76.0 yrs	(i) Hoehn and Yahr scale score, (ii) clinical examinations, and (iii) MDS-UPDRS	Baseline and End of study	The 3-month hospitalization rate for COVID-19 was 0.6% in Parkinson's disease, 3.3% in parkinsonism, and 0.7% in controls. The adjusted hazard ratio (*p*=0.006) in parkinsonism compared with controls	Parkinsonism, Parkinson's disease alone, is assumably not a risk factor for the rising hospitalization of patients with COVID-19	(i) different risk found for PD and PS suggests that this factor could have biased the results only in a small part
Raphael Scherbaum et al. [26]	2021-May	Germany	The study aimed to provide a nationwide analysis on hospitalized PD patients in Germany and evaluate the impact of the COVID-19 pandemic	Cross-sectional	Not mentioned	N (COVID-19 + PD) = 693 (419 M, 274 F), AgeM = 80.8 yrs N (COVID-19 + NOPD) = 30,179 (16,373 M, 13806 F), AgeM = 67.4 yrs	(i) Hoehn and Yahr scale score (ii) clinical examinations (iii) NMSS (iv) Hoehn and Yahr scale	Assessment during COVID-19	COVID-19 frequency was significantly higher in the population of 64,434 PD patients than in non- PD patients. Especially in subjects with advanced age (≥65 years); COVID-19 inpatient mortality rate was much higher in PD patients than in non-PD patients (*p* < 0.001)	Patients with PD are more frequently influenced by COVID-19 and sorrow from increased COVID-19 associated mortality than non-PD patients	(i) A risk of selection bias (ii) limited generalizability of results

**Table 3:** Key results of papers (study population = persons with PD).

Contribution	Year	Country	Aim of the study	Study design	PD duration	Sample size	Survey tool	Assessment time	Outcomes: Self-reported changes on symptoms in patients without COVID-19	Main results	Limitations
Fabbri et al. [27]	2021-July	France	To explore the outcomes of the first COVID-19 lockdown on motor and nonmotor symptoms in a cohort with Parkinson's disease	Cross-sectional	Mean = 8.67 yrs	*N* = 2563, EG1 (PEC group) = 441(242 M, 199 F), AgeM = 66.84 yrs, EG2 (community-based group) = 2212 (1271 M, 941 F), and AgeM = 68.37 yrs	(i) PGI-I (ii) PDQ-8	(i) Survey after lockdown: 20th April to 16th May 2020	(i) Worsened clinical symptoms 40.9%, (ii) worsened pain (9.2%), rigidity (9.0%), tremor (8.4%), (iii) walking troubles (6.9%), Akinesia (6.8%), anxiety (6.1%), fatigue (5.4%), and (iv) balance disturbances (4.5%) and sleep disorders (4.1%)	The first COVID-19 lockdown harmed the motor and nonmotor symptoms in patients with PD, and actions should be created to prevent interruptions in care, including physiotherapy, physical activity, and telemedicine	(i) Failure to conduct face-to-face polls (ii) Missing data on follow-up by patients (iii) Absence of patients with severe Parkinson's in the study
Beatrice Ana-Maria Anghelescu et al. [8]	2021-Aug	Canada	Comprehending the consequences of COVID-19 on patients' clinical symptoms of PD in both motor and nonmotor aspects and learning from the unpretentious experiences of virtual therapy from PD patients could support	Qualitative research	Not mentioned	*N* = 22 (16 M, 6 F) and AgeM = 70.5 yrs	(i) Interview (qualitative analysis)	During pandemic: 28th April to 13th May 2020	(i) Activities of daily living; attitudes and perceptions, (ii) worsening in motor including increased OFF-times, tremors/shaking, and stuttering, and (iii) worsening in nonmotor symptoms	This study provides an overview of the experiences of PD patients during COVID-19 with an emphasis on the clinical and remote care aspects. The three main issues reported by patients with PD as a result of the epidemic were the effects on clinical care, personal life, and changes in attitudes and perceptions	(i) Used a preselected sample (selection bias) and (ii) due to the restrictions and suspension of in-person clinical visits, they were not able to perform any clinical assessments such as MDS-UPDRS at the time
Marika Falla et al. [28]	2021-July	Italy	To acquire data on the consequences of lockdown efforts on motor and nonmotor symptoms of PD enrolled in a research project and to evaluate the feasibility of a remote, web-based video evaluation for PD patients during the COVID-19 pandemic	Quasiexperimental	Mean = 5.7 yrs	*N* = 14 (7 M, 7 F) and AgeM = 64.9 yrs	(i) MDS- UPDRS; (ii) UPDRS; (iii) AES-S, AES-I; (iv) LSNS-R; (v) PDQ-39-SI; (vi) FES-I; (vii) ABCs-I	Baseline: February 2020 Follow-up: 24th April to 1st May 2020	(i) Worsened nonmotor aspects (MDS-UPDRS part I) (*p* < 0.001), (ii) worsened motor and nonmotor symptoms (MDS-UPDRS total score) (*p*=0.023), (iii) worsened OR-PAS (Parkinson anxiety scale) avoidance behaviour (*p* < 0.001), and (iv) worsened OR-PAS total (Parkinson anxiety scale) (*p* < 0.007)	The findings indicate impaired nonmotor symptoms in patients with PD and recommend the feasibility and use of telemedicine in monitoring patients with PD during the COVID-19 epidemic	(i) Limited number of patients and (ii) MDSUPDRS, which is derived from UPDRS, has not yet been validated remotely and/or used for comparison between incline and telemedicine visit scores
Alessandra Dodich et al. [29]	2021-March	Italy	To consider the role of social-cognitive capacities in the perceived effect of COVID-19 emergency and the consequences of lockdown measures on patients' social networks and caregivers' commitment	Quasiexperimental	Mean = 5.7 yrs	*N* = 14 (7 M, 7 F) and AgeM = 55.5 yrs	(i) MDS-UPDRS; (ii) IRI; (iii) LSNS-R (iv) CBI	Baseline: February 2020 follow-up: 20th April to 3rd May 2020	(i) Patients showed high levels of IRI personal distress, (ii) patients felt the disease went faster in the 2 months of lockdown, (iii) patients reported increased memory difficulties, (iv) increased anxiety was reported, and (v) worsened motor functioning	Patients with social cognition disorders displayed a remarkably lower apprehension about the possible effects of COVID-19 on their health. During the lockdown, the burden on caregivers and patients' social networks remained constant. These results suggest that PD sociocognitive disorders may affect patients' ability to estimate the effects of COVID-19 infection. The lack of significant growth in caregiver burden and social isolation	(i) Small sample size and the lack of a control group and (ii) inability to carefully assess the effects of individual sociodemographic and psychological factors
Delfina Janiri et al. [30]	2020-Nov	Italy	To determine risk/protective factors associated with subjective worsening of psychiatric symptomatology during COVID-19	Cross-sectional	Not mentioned	*N* = 101 (43 M, 58 F) and AgeM = 71.86 yrs	(i) MDS-UPDRS; (ii) H and Y stage; (iii) subjective worsening of neurological symptoms during the COVID-19 outbreak	During pandemic: 1st to 15th April 2020	(i) Worsening of psychiatric symptomatology 23 subjects, (ii) the most frequent symptom was depression, followed by insomnia, and (iii) worsening of neurological symptoms (*p*=0.001) and lifetime irritability	The results demonstrated that the stress of COVID-19 outbreaks might have a more significant impact on people with PD and people with a history of lifelong psychiatric symptoms. Interventions desired to reduce mood irritability can indirectly influence the health of patients with PD	—
Roberta Baschi et al. [10]	2020-Dec	Italy	To consider the motor, cognitive, and behavioral changes during the COVID-19 lockdown in patients with Parkinson's disease (PD) with and without mild cognitive impairment	Cross-sectional	Not mentioned	*N* = 96 (58 M, 38 F), AgeM = 67.3 yrs PD-NC = 34(23 M, 11 F), AgeM = 65.4 yrs PD-MCI = 31(20 M, 11 F), AgeM = 66.7 yrs MCInoPD = 31(15 M, 16 F), AgeM = 70 yrs	(i) NPI (ii) MDS-UPDRS	10 weeks after the COVID-19 lockdown: 20th to 30th May 2020	(i) Worsening of cognitive, pre-existing, and new behavioral symptoms, and motor symptoms. (ii) A significantly higher frequency of cognitive impairment (*p*=0.034), fatigue (*p*=0.036), and speech (*p*=0.013) than PD-NC. (iii) PD-MCI showed significantly higher frequencies in several MDS-UPDRS items compared to MCInoPD, particularly regarding pain (*p*=0.001), turning in bed (*p*=0.006), getting out of bed (*p*=0.001), and walking and balance (*p*=0.003)	COVID-19 lockdown will worsen cognitive, behavioral, and motor symptoms in people with PD and MCI, especially PD-MCI	(i) Small sample size
Birgul Balci et al. [9]	2021-June	Turkey	To reach the physical activity and anxiety-depression levels between Parkinson's disease patients and controls during the lockdown	Cross-sectional	Mean = 8 yrs	*N* = 88, PD = 45 (30 M, 15 F), AgeM = 67 yrs, Control = 43 (24 M, 19 F), and AgeM = 66 yrs	(i) PASE (ii) HADS via telephone interview	After lockdown: 15th to 20th June 2020	(i) Worsened motor and nonmotor symptoms: tremor 11 out 45, dyskinesia 7 out 45, bradykinesia (slowness of movements such as turning in bed and rising of chair) 22 out 45, rigidity 18 out 45, gait impairments (height of foot lift, stride length/speed, arm swing) 18 out 45, freezing of gait 7 out 45, balance problem 15 out 45, cognitive impairment (paying attention, following conversations) 7 out 45, sleep problems 9 out 45, daytime sleepiness 7 out 45, pain and other sensations (such as aches, cramps, and tingling) 15 out 45	Lockdown may increase the motor and nonmotor symptoms of Parkinson's disease associated with physical inactivity	(i) The information was obtained with self-reported questionnaires, which might increase the risk of bias and insufficient recall
Fukiko Kitani-Morii et al. [31]	2021-Jan	Japan	To elucidate the effect of social restrictions imposed due to the COVID-19 pandemic on neuropsychiatric symptoms in PD patients and to identify risk factors associated with these signs	Cross-sectional	Mean = 5 yrs	*N* = 71, PD = 39 (25 M, 14 F), AgeM = 74.7 yrs, CG = 32 (5 M, 27 F), and AgeM = 71.56 yrs	(i) MDS-UPDRS	During pandemic: 22th April to 15th May 2020	(i) Clinical depression (PHQ-9 score) were more in PD patients (39%) than controls (6%) (*p*=0.002). (ii) MDS-UPDRS part 2 score was correlated with the presence of clinical depression (PHQ-9 score) (*p*=0.025) and clinical anxiety (*p*=0.013)	Patients with Parkinson's are more likely to develop depression than others in social stress, such as an epidemic	(i) Absence of information about depression, anxiety, and insomnia of participants before the COVID-19 pandemic and a female bias in sex ratio in the control group
Elisa Montanaro et al. [32]	2021-May	Italy	To investigate the psychological impact of the COVID-19 outbreak on APD patients and their caregivers by assessing distress, worries, depressive, and anxious symptoms; also, hypothesized an increasing influence of the COVID-19 outbreak on PD patients and their caregivers	Quasiexperimental	Mean = 13.4 yrs	*N* = 100 (60 M, 40 F) and AgeM = 62.4 yrs	(i) Telephone interview: HADS-A; HADS-D	During lockdown (T0): April to May 2020 After the end of the lockdown in Italy (T1): June to August 2020	(i) Depression was observed in 35% of APD patients and anxiety in 39%, with a significant reduction of the latter after the lockdown (*p*=0.023) (ii) patients' main worries were: a possible higher risk of COVID-19 infection (25%), interruption of nonpharmacological treatments (35%), interruption of outpatient clinics (38%), PD complications related to COVID-19 (47%)	The findings show that anxiety, worry, and special needs are more prevalent in Parkinson's patients during the epidemic	(i) Lack of baseline evaluations of anxiety and depression before the COVID-19 outbreak (ii) Our cohort focused mostly on APD patients treated with DAT
Galit Yogev-Seligmann et al. [33]	2021-July	Israel	To characterize the effects of COVID-19 social distancing on the function, health, and well-being of people with Parkinson's disease (PD) and test the association of these effects	Cross-sectional	Mean = 10.6 yrs	*N* = 142 (83 M, 59 F) and AgeM = 70.6 yrs	(i) Web-based survey: Part 1 : 27 multiples-choice questions regarding changes in function, health, medical care, and well-being; Part 2 consisted of the PAM-13	After lockdown: 10th May to 1st June 2020	(i) Deteriorated walking ability 37%, increased need for assistance with ADL 24%. Only 4.3% reported falling more often than before the lockdown, (ii) worsened of disease symptoms 53%, while 33.8% reported weight gain. 83.2% were able to control other comorbidities; 34.5% reported an overall worsening of their disease condition, and (iii) feeling more tired 43%, and 42.1% increases in at least one of the following: depression, anxiety, loneliness, or worry about the future. 26.1% reported a lack of spousal support during the lockdown	Respondents reported a decline in various functions, health, and well-being. Rehabilitation stopped for more than 60% of people. Among those who reported deteriorating health, the majority attributed this to stopping rehabilitation treatments or reducing physical activity	(i) Lack of generalization of findings to the whole community for reasons such as spouses' support of patients, regular participation in physical activities, and computer literacy
Megan Feeney et al. [5]	2021-Jan	USA	To experiment with the social and emotional effect of the pandemic and social distancing on PwPD and to examine the factors contributing to accessing alternative health care mechanisms	Cross-sectional	Mean = 7.0 yrs	*N* = 1342 (656 M, 679 F, other 1, missing 6) and AgeM = 70.9 yrs	(i) Online questionnaire	During pandemic: 13th May to 11 June 2020	(i) Approximately half of respondents reported a negative change in PD symptoms, with 45–66% reporting mood disturbances. (ii) Almost half of PwPD reported reduced hours of exercise, and a majority of respondents reported a reduction in activities outside of their residence	Symptoms of PD and its management practices were significantly affected by COVID-19. Finally, it is essential to expand the use of telehealth, especially to reach disadvantaged and low-income populations	—
Anouk van der Heide et al. [34]	2020-Aug	Netherlands	The main aim is to test the hypothesis that COVID-19 pandemic has led to an increased psychological distress in PD, worsening PD symptoms	Cross-sectional	Mean = 3.9 yrs	*N* = 358 (220 M, 138 F) and AgeM = 62.8 yrs	(i) MDS-UPDRS-I and MDS-UPDRS-II	Baseline and end of study: assessment at the last PPP visit: 7.5 months prior to COVID-19 survey Assessment during COVID-19 survey: on average 1.4 months COVID-19	Worsened in frigidity: 42.6% Worsened in fatigue: 41.5% Worsened in tremor: 40.7%: Worsened in pain: 33.8% Worsened in concentration: 32.5% Worsened in memory problems: 31.9% Worsened in depressed mood: 31.6% Worsened in gait and freezing = 31.5%	The main results show that stress caused by the COVID-19 epidemic leads to worsening of PD symptoms by provoking psychological distress as well as lifestyle changes	(i) The impact of COVID-19 might well be even more dramatic for patients with late-stage PD; (ii) due to the retrospective nature of the survey, responses might be less accurate, since for most questions they had no baseline data
Keisuke Suzuki et al. [35]	2021-Mar	Japan	Researchers investigated changes in motor symptoms, cognition, mood, sleep, and stress as well as determinants of QOL in PD patients during the COVID-19 outbreak	Cross-sectional	Mean = 5.8 yrs	*N* = 100 (45 M, 55 F) and AgeM = 72.2 yrs	(i) HADS-A; HADS-D (ii) PCS (iii) MCS scores of the SF-8	Assessment during COVID-19 survey	Worsened in tremor: 34.1% Worsened in rigidity: 40.4% Worsened in bradykinesia: 20.2% Worsened in gait: 55.0% Worsened in postural stability: 43.0% Worsened in cognition: 34.0% Worsened in mood: 36.0% Worsened in sleep:34.0%	In PD patients, gait disturbance, rigidity, disease severity, smoking, levodopa equivalent dose, weight loss, anxiety, depression, female sex, stress, and disease duration worsened	(i) Selection bias cannot be ruled out. (ii) No baseline data from the period before the COVID-19 pandemic. (iii) Motor symptoms after the outbreak of COVID-19 were not assessed a clinical examination
Tommaso Schirinzi et al. [36]	2020-Aug	Italy	This study aimed to examine the changes in physical activity in PD patients due to COVID-19	Cross-sectional	Mean = 6.5 yrs	*N* = 74 (37 M, 37 F) and AgeM = 61.3 yrs	(i) IPAQ-SF (ii) PWBM (iii) BDI	Assessment during COVID-19 survey: April 20, 2020 and May 2, 2020	Patients with PD worsened, 55.5% in motor and nonmotor symptoms, 11.3% in depression, lower total MET (1714) min/week	The majority of patients reported that their condition had worsened during lockdown, which was mainly due to reduced physical activity	(i) The lack of objective and accurate measurement of clinical features. (ii) The clinical outcome consisted just in the individual perception of patients
Rosa De Micco et al. [37]	2020- Nov	Italy	Research team assessed the psychological impact of the 40-day lockdown in a large cohort of patients with Parkinson's disease	Cross-sectional	Mean = 3.26 yrs	*N* = 94 (62 M, 32 F), AgeM = 63.98 yrs	(i) Event scale-revised; (ii) Kessler psychological distress scale; (iii) NMSS (iv) PDQ-39	Assessment at the last PPP visit: 6 months prior to COVID-19 survey Assessment during COVID-19 survey: a mean of 40 days of lockdown	Worsened in sleep/fatigue: 33% Worsened in mood/cognition: 40% Worsened in attention/memory: 46% Worsened in perceptual problems: 21%	They recognized specific PD motor and nonmotor characteristics predisposing them to the higher psychological impact of stressful circumstances	(i) Other factors may have affected the psychological well-being of PD cohort during the lockdown period. (ii) More solid conclusions about the pathophysiological processes potentially underlying our results
Aline Nogueira Haas et al. [38]	2022-Jan	Brazil	(1) To assess mental health symptoms in people with Parkinson's (PwP) in self-isolation, before and during the COVID-19 pandemic; (2) to investigate associations between mental health and physical activity levels	Cross-sectional	Mean = 7.29 yrs	*N* = 156 (78 M, 78 F) and AgeM = 64 yrs	(i) An online self-administered and validated questionnaire	2 months after the pandemic: 12th to 21st of May 2020	(i) Worsened mental health symptoms over the time: anxiety (*p* < 0.001), fear (*p* < 0.001], and thoughts of death (*p*=0.001]. (ii) A lower physical activity level during the pandemic is related to increased probability of thoughts of death. (iii) Anxiety, fear and depression were not associated with physical activity levels	Anxiety, fear, and thoughts of death worsened during the COVID-19 pandemic. Lower physical activity level during the pandemic was related to an increased probability of thoughts of death	(i) The small number of responses obtained from the midwest and northeast Brazilian regions; (ii) all data were self-reported potentially suffering from social desirability bias; (iii) prepandemic variables were assessed retrospectively and could suffer from recall bias
Guo et al. [39]	2020-Oct	China	To examine the effect of lockdown during the COVID-19 pandemic	Quasiexperimental	Mean = 6.05 yrs	*N* = 113 (66 M, 47 F) and AgeM = 69.5 yrs	(i) COVID-19 Questionnaire (ii) PDQ-39	(i) During: 1st February to 31th March, 2020 (ii) After: 1st April to 30th April, 2020	(i) Worsening of PD symptoms; (ii) increased tremor (39.8%), increased stiffness (60.2%), increased slowness (60.2%), newly appearing or worsening of dyskinesia (39.8%), newly appearing or worsening of fluctuation of symptoms (40.7%), excessive fatigue (29.6%), feeling/appear stressed or anxious (79.6%), feeling/appear depressed (50.0%), sleep disorders (39.8%), reduced appetite (10.2%), and increased aches and pains (29.6%)	Quality of life for patients during the period of epidemic prevention and control was worse than that after epidemic prevention and control	—

**Table 4:** Key results of papers (study population = persons with COVID-19 and PD, and non-COVID-19 and PD population).

Contribution	Year/month	Country	Aim of the study	Study design	Study population	PD duration	Sample size	Survey tool	Assessment time	Outcomes: in-hospital consequences (morbidity or severe COVID-19)	Outcomes: Self-reported changes on symptoms in patients without COVID-19	Main results	Limitations
Eleonora Del Prete et al. [37]	2020-Sep	Italy	To estimate the prevalence, mortality, and case-fatality of COVID-19 and to explore the presence of risk factors for COVID-19 in PD patients; also, to investigate the effects of lockdown on motor and nonmotor symptoms	Case control	Both of them	Mean = 8.93 yrs	*N* = 740 PD without COVID-19 = 733, AgeM = 75.05 yrs PD with COVID = 7 (4 M, 3 F), and AgeM = 75.51 yrs	(i) Interview (ii) DASS-21	During lockdown: 10th April to 4th May 2020	(i) Higher mortality (0.13%) than Italy and in Tuscany but in line with national data. (ii) Higher case-fatality (14%) than regional data but in line with national data. (iii) Higher prevalence of COVID-19 (0.9%) in PD than the national and regional. (iv) Higher hypertension (*p* < 0.001) and diabetes (*p*=0.049)	(i) Worsened in parkinsonian symptoms (29.6%). (ii) Worsened in Mmod (24.7%). (iii) Worsened in anxiety (25.0%). (iv) Worsened in insomnia (22.2%)	A higher prevalence of COVID-19 was marked in the PD population, but it was not clear whether PD alone was a risk factor for COVID-19. Hypertension and diabetes were determined as risk factors for COVID-19 in PD. PD patients did not experience worsening mental symptoms	(i) the small number of COVID-19 patients (ii) Longer follow up is need
Ethan Brown et al. [38]	2020-Oct	USA	First: to comprehend the symptoms and outcomes of SARS-CoV-2 infection in people with and without PD Second: to specify the effects of COVID-19 on motor and nonmotor symptoms Third: to comprehend the consequences of the COVID-19 and associated public health measures on people with and without PD, even if not directly infected with SARS-CoV-2	Cross-sectional	Both of them	Mean = 5.11 yrs	*N* = 6881 COVID-19 + PD = 51(24 M, 27 F), AgeM = 65 yrs COVID-19 + NoPD = 26(2 M, 24 F), AgeM = 57 yrs NoCOVID-19 + PD = 5378(2780, 2598F), AgeM = 68 yrs NoCOVID-19 + NOPD = 1426(311 M, 1115F), and AgeM = 61 yrs	(i) Survey by online study FI: NMSQ	During pandemic: 23th April to 23th May 2020	(i) Worsening of existing hyposmia was reported of people with PD and none of those without PD; (ii) new motor symptoms were reported and reported worsening of at least one existing motor symptom; (iii) new and worsening nonmotor symptoms were reported for all domains: mood, cognition (41% worsening), sleep (12% new, 59% worsening), and autonomic (7.8% new, 29% worsening); (iv) people with PD and COVID-19 experienced new or worsening motor and nonmotor symptoms	(i) Behavioral and environmental risk factors for COVID-19 were more common in people without PD than with PD. Reported disrupted medical care, exercise, and social activities and worsened motor and nonmotor symptoms	For people with PD without COVID-19, disturbances in medical care, essential daily activities, exercise, and social activities were frequently seen, causing motor and nonmotor symptoms	(i) Response rate was moderate, (ii) survey completion was naturally limited to people who were healthy enough to log-in online and fill out a survey, and (iii) certain populations were underrepresented, and the fact that we did see significantly greater disruption from the pandemic in some of these groups

**Table 5:** Distribution of papers by journals, quartile scores, and IF.

Journal name	Column labels			Grand total
Row labels	Q1	Q2	Q3	
Movement Disorders IF = 10.33	7			7
Journal of Parkinson's Disease IF = 5.56	3			3
Journal of Neurology IF = 4.84	3			3
Frontiers in Psychiatry IF = 4.15		2		2
PLoS ONE IF = 3.24	2			2
Neurological Sciences IF = 3.3		2		2
Geriatrics Without IF			1	1
Parkinson's Disease IF = 2.7		1		1
Frontiers in Psychology IF = 2.99		1		1
New Zealand Medical Journal Without IF		1		1
International Journal of Rehabilitation Research IF = 1.47		1		1
NPJ Parkinson's Disease IF = 8.65	1			1
Journal of Neural Transmission IF = 3.57	1			1
Parkinsonism and Related Disorders IF = 4.89	1			1
Acta Neurologica Belgica IF = 2.39			1	1
Sport Sciences for Health Without IF			1	1
BMC Neurology IF = 2.47			1	1
Canadian Journal of Neurological Sciences IF = 2.1			1	1
Grand total	18	8	5	31

**Table 6:** Worsened symptoms in Parkinson's patients who have been affected in the pandemic.

Worsened symptoms	References																			Total
	[21]	[22]	[23]	[24]	[25]	[26]	[27]	[28]	[29]	[30]	[5]	[39]	[32]	[33]	[34]	[35]	[36]	[37]	[38]	
1. Abnormalities of movement (motor functioning)	+			+		+								+			+	+	+	7
1.1. Tremor/shaking	+	+	+				+					+	+				+			7
1.2. Walking troubles	+					+				+				+						4
1.3. Gait and balance disturbances	+					+	+					+	+							5
1.4. Bradykinesia						+	+						+				+			4
1.5. Dyskinesia						+	+										+			3
1.6. Rigidity (frigidity)	+						+					+	+				+			5
1.7. Falling										+										1
1.8. Fatigue	+					+				+		+			+		+			6
1.9. Postural stability													+							1
2. Nonmotor functioning (psychiatric symptomatology)		+			+								+						+	4
2.1. Pain	+	+				+						+					+			5
2.2. Sensations (such as aches, cramps, and tingling)							+	+												2
2.3. Distress/depression				+	+			+	+	+		+		+			+			8
2.4. Akinesia	+																			1
2.5. Anxiety and stress	+			+				+	+	+						+	+	+		8
2.6. Cognitive dysfunction						+	+								+					3
2.7. Sleep disorders (insomnia)	+						+						+		+		+	+		6
2.8. Speech disorders (stuttering)		+				+														2
2.9. Perceptual problems	+																			1
2.10. Fear and depression				+												+				2
2.11. Appetite																	+			1
2.12. Memory difficulties and concentration				+								+			+					3
2.13. Loneliness or worry									+	+									+	3
2.14. Increased need for assistance										+										1
2.15. Mood disturbances (anger, irritability, and frustration)					+						+		+		+		+	+		6
Negative impact of the pandemic in Parkinson's patients (yes or no)	Yes	Yes	Yes	Yes	Yes	Yes	Yes	Yes	Yes	Yes	Yes	Yes	Yes	Yes	Yes	Yes	Yes	No	Yes	Yes: 1, No: 1

**Table 7:** Symptoms and consequences of Parkinson's patients with COVID-19.

Worsened symptoms	References													Total
	[10]	[11]	[12]	[13]	[40]	[15]	[16]	[17]	[18]	[19]	[20]	[37]	[38]	
1. Abnormalities of movement (motor functioning)		+		+					+				+	4
1.1. Tremor/shaking				+										1
1.2. Walking troubles				+										1
1.3. Gait and balance disturbances				+										1
1.4. Bradykinesia				+										1
1.5. Dyskinesia				+										1
1.6. Rigidity (frigidity)				+										1
1.7. Falling				+										1
1.8. Disability									+					1
1.9. Dystonia				+										1
1.10. Freezing				+										1
2. Nonmotor functioning (psychiatric symptomatology)				+					+				+	3
2.1. Sensations (such as aches, cramps, and tingling)				+										1
2.2. Thought disorders				+										1
2.3. Distress/depression				+										1
2.4. Anxiety and stress				+										1
2.5. Cognitive dysfunction									+				+	2
2.6. Sleep disorders (insomnia)				+					+					2
2.7. Speech disorders (stuttering)				+										1
2.8. Mentation				+										1
2.9. Mood disturbances (anger, irritability, and frustration)		+							+				+	3
2.10. Dementia					+			+						2
3. Clinical outcomes (pain and cough)														—
3.1. Pain	+		+	+					+					4
3.2. Anorexia						+			+					2
3.3. Fatigue									+					1
3.4. Weakness		+												1
3.5. Fever	+		+						+					3
3.6. Hypertension											+	+		2
3.7. Cough	+					+			+					3
3.8. Dyspnea	+								+					2
3.9. Headache	+													1
3.10. Constipation				+										1
3.11. Diarrhea									+					1
3.12. Smell loss									+				+	2
3.13. Vitamin D deficiency											+			1
3.14. Urinary disorders				+					+					2
3.15. Cardiac disease											+			1
3.16. Cerebrovascular disease											+			1
3.17. Diabetes											+	+		2
3.18. Kidney disease											+			1
3.19. Flu												+		1
3.20. Lung disease													+	1
4. Activities of daily living														—
4.1. Salivation				+										1
4.2. Hand writing				+										1
4.3. Dressing				+										1
4.4. Turning in bed				+										1
4.5. Swallowing				+										1
4.6. Cutting food				+										1
4.7. Hygiene				+										1
4.8. Autonomic													+	1
5. Other outcomes														—
5.1. Hospitalization		+												1
5.2. Mortality rate		+					+				+	+		4
5.3. Prevalence												+		1
5.4. Case fatality												+		1
PD is risk factor (yes or no)	No	Yes	No	No	No	Yes	No	No	Yes	Yes	Yes	Unclear	Yes	Yes: 6 No: 6 Unclear: 1

## Data Availability

All data generated or analyzed during this study are included within the article.

## Abbreviations

MDS-UPDRS: Motor symptoms: unified Parkinson's disease rating scale
PDQ-8: Parkinson's disease questionnaire
PGI-I: Patient global impression of improvement
PDQ-39: Parkinson's disease questionnaire
PDMSS: PD motor symptom scale
PSS-10: Perceived stress scale
PALQ: Physical activity levels questionnaire
UPDRS: The modified Hoehn and Yahr scale and unified PD rating scale
DASS-21: Depression anxiety stress scale-21 item
AES-S, AES-I: Apathy evaluation scale
LSNS-R: Lubben social network scale-revised
PDQ-39: Parkinson's disease questionnaire
PDQ-39-SI: With summary index
FES-I: Falls efficacy scale international
ABCs-I: The activity-specific balance confidence scale
IRI: Empathic attitude: interpersonal reactivity index
CBI: Caregivers' burden caregiver burden inventory
H and Y stage: Hoehn and Yahr stage
NPI: Neuropsychiatric inventory
PASE: Physical activity scale for the elderly
HADS-A; HADS-D: Hospital anxiety and depression scale
FI: Fox insight
NMSQ: Nonmotor symptoms questionnaire
PAM-13: Patient activation measure
PCS: Physical component summary
MCS: Mental component summary
(SF)-8: Scores of the short form
IPAQ-SF: International physical activity questionnaires-short form
PWBM: Parkinson's well-being map
BDI: Beck depression index
NMSS: 12-item Zarit burden inventory nonmotor symptom scale
CISI-PD: Clinical impression of severity index for Parkinson's disease
PD-MCI: Parkinson's disease with mild cognitive impairment
MCInoPD: Mild cognitive impairment not associated with Parkinson's disease
PD: Parkinson disease
PD-NC: Parkinson's disease with normal cognition
CG: Control group
EG: Experimental group
AgeM: Age median
PEC: Parkinson expert center
SMT: Standard medical treatment
APD: Advanced Parkinson's disease.

## References

[1] Wang H., Liu Y., Zhao J., Guo X., Hu M., Chen Y. (2021). Possible inflammatory mechanisms and predictors of Parkinson’s disease patients with fatigue (Brief Review). *Clinical Neurology and Neurosurgery*.

[2] Tysnes O. B., Storstein A. (2017). Epidemiology of Parkinson’s disease. *Journal of Neural Transmission*.

[3] Ou Z., Pan J., Tang S. (2021). Global trends in the incidence, prevalence, and years lived with disability of Parkinson’s disease in 204 countries/territories from 1990 to 2019. *Frontiers in Public Health*.

[4] Blakemore R., Pascoe M., Horne K.-L. (2021). Higher perceived stress and exacerbated motor symptoms in Parkinson’s disease during the COVID-19 lockdown in New Zealand. *The New Zealand Medical Journal*.

[5] Feeney M. P., Xu Y., Surface M. (2021). The impact of COVID-19 and social distancing on people with Parkinson’s disease: a survey study. *NPJ Parkinson’s disease*.

[6] Antonini A., Leta V., Teo J., Chaudhuri K. R. (2020). Outcome of Parkinson’s disease patients affected by COVID‐19. *Movement Disorders*.

[7] Fearon C., Fasano A. (2021). Parkinson’s disease and the COVID-19 pandemic. *Journal of Parkinson’s Disease*.

[8] Anghelescu B. A., Bruno V., Martino D., Roach P. (2022). Effects of the COVID-19 pandemic on Parkinson’s disease: a single-centered qualitative study. *The Canadian Journal of Neurological Sciences/Journal Canadien des Sciences Neurologiques*.

[9] Balci B., Aktar B., Buran S., Tas M., Donmez Colakoglu B. (2021). Impact of the COVID-19 pandemic on physical activity, anxiety, and depression in patients with Parkinson’s disease. *International Journal of Rehabilitation Research*.

[10] Baschi R., Luca A., Nicoletti A. (2020). Changes in motor, cognitive, and behavioral symptoms in Parkinson’s disease and mild cognitive impairment during the COVID-19 lockdown. *Frontiers in Psychiatry*.

[11] Brown E. G., Chahine L. M., Goldman S. M. (2020). The effect of the covid-19 pandemic on people with Parkinson’s disease. *Journal of Parkinson’s Disease*.

[12] van Wamelen D. J., Leta V., Johnson J. (2020). Drooling in Parkinson’s disease: prevalence and progression from the non-motor international longitudinal study. *Dysphagia*.

[13] Boika A. V., Sialitski M. M., Chyzhyk V. A., Ponomarev V. V., Fomina E. G. (2021). Post‐COVID worsening of a Parkinson’s disease patient. *Clinical Case Reports*.

[14] Cilia R., Bonvegna S., Straccia G. (2020). Effects of COVID‐19 on Parkinson’s disease clinical features: a community‐based case‐control study. *Movement Disorders*.

[15] Zhang X., Tan R., Lam W. C. (2020). PRISMA (preferred reporting items for systematic reviews and meta-analyses) extension for Chinese herbal medicines 2020 (PRISMA-CHM 2020). *The American Journal of Chinese Medicine*.

[16] Polmann H., Domingos F. L., Melo G. (2019). Association between sleep bruxism and anxiety symptoms in adults: a systematic review. *Journal of Oral Rehabilitation*.

[17] Buccafusca M., Micali C., Autunno M., Versace A. G., Nunnari G., Musumeci O. (2021). Favourable course in a cohort of Parkinson’s disease patients infected by SARS-CoV-2: a single-centre experience. *Neurological Sciences*.

[18] de Marcaida J. A., Lahrmann J., Machado D. (2020). Clinical characteristics of coronavirus disease 2019 (COVID-19) among patients at a movement disorders center. *Geriatrics*.

[19] Sorbera C., Brigandì A., Cimino V. (2021). The impact of SARS-COV2 infection on people in residential care with Parkinson Disease or parkinsonisms: clinical case series study. *PLoS One*.

[20] Xu Y., Surface M., Chan A. K. (2021). COVID-19 manifestations in people with Parkinson’s disease: a USA cohort. *Journal of Neurology*.

[21] Sainz-Amo R., Baena-Álvarez B., Pareés I. (2021). COVID-19 in Parkinson’s disease: what holds the key?. *Journal of Neurology*.

[22] Zhai H., Lv Y., Xu Y. (2021). Characteristic of Parkinson’s disease with severe COVID-19: a study of 10 cases from Wuhan. *Journal of Neural Transmission*.

[23] Parihar R., Ferastraoaru V., Galanopoulou A. S., Geyer H. L., Kaufman D. M. (2021). Outcome of hospitalized Parkinson’s disease patients with and without COVID‐19. *Movement Disorders Clinical Practice*.

[24] Nwabuobi L., Zhang C., Henchcliffe C. (2021). Characteristics and outcomes of Parkinson’s disease individuals hospitalized with COVID‐19 in a New York city hospital system. *Movement disorders clinical practice*.

[25] Vignatelli L., Zenesini C., Belotti L. M. (2021). Risk of hospitalization and death for COVID‐19 in people with Parkinson’s disease or parkinsonism. *Movement Disorders*.

[26] Scherbaum R., Kwon E. H., Richter D. (2021). Clinical profiles and mortality of COVID‐19 inpatients with Parkinson’s disease in Germany. *Movement Disorders*.

[27] Fabbri M., Leung C., Baille G. (2021). A French survey on the lockdown consequences of COVID-19 pandemic in Parkinson’s disease. The ERCOPARK study. *Parkinsonism and Related Disorders*.

[28] Falla M., Dodich A., Papagno C. (2021). Lockdown effects on Parkinson’s disease during COVID-19 pandemic: a pilot study. *Acta Neurologica Belgica*.

[29] Dodich A., Papagno C., Turella L. (2021). The role of social cognition abilities in Parkinson’s disease in the era of COVID-19 emergency. *Frontiers in Psychology*.

[30] Janiri D., Petracca M., Moccia L. (2020). COVID-19 pandemic and psychiatric symptoms: the impact on Parkinson’s disease in the elderly. *Frontiers in Psychiatry*.

[31] Kitani-Morii F., Kasai T., Horiguchi G. (2021). Risk factors for neuropsychiatric symptoms in patients with Parkinson’s disease during COVID-19 pandemic in Japan. *PLoS One*.

[32] Montanaro E., Artusi C. A., Rosano C. (2022). Anxiety, depression, and worries in advanced Parkinson disease during COVID-19 pandemic. *Neurological Sciences*.

[33] Yogev-Seligmann G., Kafri M. (2021). COVID-19 social distancing: negative effects on people with Parkinson disease and their associations with confidence for self-management. *BMC Neurology*.

[34] Van der Heide A., Meinde M. J., Bloem B. R., Helmich R. C. (2020). The impact of the COVID-19 pandemic on psychological distress, physical activity, and symptom severity in Parkinson’s disease. *Journal of Parkinson’s Disease*.

[35] Suzuki K., Numao A., Komagamine T. (2021). Impact of the COVID-19 pandemic on the quality of life of patients with Parkinson’s disease and their caregivers: a single-center survey in tochigi prefecture. *Journal of Parkinson’s Disease*.

[36] Schirinzi T., Di Lazzaro G., Salimei C. (2020). Physical activity changes and correlate effects in patients with Parkinson’s disease during COVID‐19 lockdown. *Movement disorders clinical practice*.

[37] De Micco R., Siciliano M., Sant’Elia V. (2021). Correlates of psychological distress in patients with Parkinson’s disease during the COVID‐19 outbreak. *Movement disorders clinical practice*.

[38] Haas A. N., Passos-Monteiro E., Delabary M. D. (2022). Association between mental health and physical activity levels in people with Parkinson’s disease during the COVID-19 pandemic: an observational cross-sectional survey in Brazil. *Sport Sciences for Health*.

[39] Guo D., Han B., Lu Y. (2020). Influence of the COVID-19 pandemic on quality of life of patients with Parkinson’s disease. *Parkinson’s Disease*.

[40] Del Prete E., Francesconi A., Palermo G. (2021). Prevalence and impact of COVID-19 in Parkinson’s disease: evidence from a multi-center survey in Tuscany region. *Journal of Neurology*.

[41] Cartella S. M., Terranova C., Rizzo V., Quartarone A., Girlanda P. (2021). Covid-19 and Parkinson’s disease: an overview. *Journal of Neurology*.

[42] Putri C., Hariyanto T. I., Hananto J. E., Christian K., Situmeang R. F. V., Kurniawan A. (2021). Parkinson’s disease may worsen outcomes from coronavirus disease 2019 (COVID-19) pneumonia in hospitalized patients: a systematic review, meta-analysis, and meta-regression. *Parkinsonism and Related Disorders*.

[43] Kainaga M., Shirota Y., Kodama S., Toda T., Hamada M. (2021). Effects of the coronavirus disease 2019 pandemic on motor symptoms in Parkinson’s disease: an observational study. *Movement Disorders*.

[44] Reuter I., Engelhardt M. (2021). Effects of the first lockdown on patients with Movement disorders during the SARS-CoV-2 pandemic. *Sports Orthopaedics and Traumatology*.

[45] Dewanjee S., Vallamkondu J., Kalra R. S., Puvvada N., Kandimalla R., Reddy P. H. (2021). Emerging COVID-19 neurological manifestations: present outlook and potential neurological challenges in COVID-19 pandemic. *Molecular Neurobiology*.

[46] Fathi M., Taghizadeh F., Mojtahedi H., Zargar Balaye Jame S., Markazi Moghaddam N. (2022). The effects of Alzheimer’s and Parkinson’s disease on 28-day mortality of COVID-19. *Revue Neurologique*.

[47] Xing F., Marsili L., Truong D. D. (2022). Parkinsonism in viral, paraneoplastic, and autoimmune diseases. *Journal of the Neurological Sciences*.

[48] Sinha S., Mittal S., Roy R. (2021). Parkinson’s disease and the COVID-19 pandemic: a review article on the association between SARS-CoV-2 and *α*-s. *Journal of movement disorders*.

[49] Sahin S., Karsidag S., Cinar N. (2021). The impact of the COVID-19 lockdown on the quality of life in chronic neurological diseases: the results of a COVQoL-CND study. *European Neurology*.

[50] Rábano-Suárez P., Martínez-Fernández R., Natera-Villalba E., Pareés I., Martínez-Castrillo J. C., Alonso-Canovas A. (2021). Impulse control disorders in Parkinson’s disease: has COVID-19 related lockdown been a trigger?. *Movement disorders clinical practice*.

[51] Satheesh N. J., Salloum-Asfar S., Abdulla S. A. (2021). The potential role of COVID-19 in the pathogenesis of multiple sclerosis-A preliminary report. *Viruses*.

[52] Estiri H., Strasser Z. H., Klann J. G., Naseri P., Wagholikar K. B., Murphy S. N. (2021). Predicting COVID-19 mortality with electronic medical records. *NPJ digital medicine*.

[53] Behl T., Kumar S., Sehgal A. (2021). Linking COVID-19 and Parkinson’s disease: targeting the role of vitamin-D. *Biochemical and Biophysical Research Communications*.

[54] Salari M., Zaker Harofteh B., Etemadifar M., Sedaghat N., Nouri H. (2021). Movement disorders associated with COVID-19. *Parkinson’s Disease*.

[55] Sabetkish N., Rahmani A. (2021). The overall impact of COVID‐19 on healthcare during the pandemic: a multidisciplinary point of view. *Health Science Reports*.

[56] Hippisley-Cox J., Coupland C. A., Mehta N. (2021). Risk prediction of covid-19 related death and hospital admission in adults after covid-19 vaccination: national prospective cohort study. *Bmj*.

[57] Chasapis C. T., Georgiopoulou A. K., Perlepes S. P., Bjørklund G., Peana M. (2021). A SARS-CoV-2–human metalloproteome interaction map. *Journal of Inorganic Biochemistry*.

[58] Krzysztoń K., Mielańczuk-Lubecka B., Stolarski J. (2021). Secondary impact of COVID-19 pandemic on people with Parkinson’s disease—results of a polish online survey. *Brain Sciences*.

[59] Phokaewvarangkul O., Virameteekul S., Bhidayasiri R. (2021). Parkinsonism hyperpyraexia syndrome in Parkinson’s disease patients undergoing deep brain stimulation: an indirect consequence of COVID-19 lockdowns. *Parkinsonism and Related Disorders*.

[60] Khoshnood R. J., Zali A., Tafreshinejad A. (2022). Parkinson’s disease and COVID-19: a systematic review and meta-analysis. *Neurological Sciences*.

[61] Guan W. J., Ni Z. Y., Hu Y. (2020). Clinical characteristics of coronavirus disease 2019 in China. *New England Journal of Medicine*.

[62] Witt V. D., Baur G., Ecke J., Kirchner A., Hauptmann B. (2021). Parkinson’s patients situation during the SARS CoV-2 pandemic and their interest in telemedicine A cross-sectional study. *PLoS One*.

[63] Buchwitz T. M., Maier F., Greuel A. (2021). Pilot study of mindfulness training on the self-awareness of motor symptoms in Parkinson’s disease–A randomized controlled trial. *Frontiers in Psychology*.

[64] Wang Y., Yang Y., Ren L., Shao Y., Tao W., Dai X. J. (2021). Preexisting mental disorders increase the risk of COVID-19 infection and associated mortality. *Frontiers in Public Health*.

[65] Lee M. Y., Oh B. M., Seo H. G. (2021). Prolonged dysphagia after a COVID-19 infection in a patient with Parkinson disease. *American Journal of Physical Medicine and Rehabilitation*.

[66] Drelich-Zbroja A., Cheda M., Kuczyńska M., Dąbrowska I., Kopyto E., Halczuk I. (2022). Parkinson’s disease in light of the COVID-19 pandemic. *Brain Sciences*.

[67] Leta V., Rodríguez‐Violante M., Abundes A. (2021). Parkinson’s disease and post–COVID‐19 syndrome: the Parkinson’s long‐COVID spectrum. *Movement Disorders*.

[68] Scorza F. A., Finsterer J., Fiorini A. C. (2021). Is SARS-CoV-2 responsible for relapses of Parkinson’s disease?. *The Egyptian Journal of Neurology, Psychiatry and Neurosurgery*.

[69] Knapik A., Szefler-Derela J., Wasiuk-Zowada D., Siuda J., Krzystanek E., Brzęk A. (2021). Isolation related to the COVID-19 pandemic in people suffering from Parkinson’s disease and activity, self-assessment of physical fitness and the level of affective disorders. *Healthcare*.

[70] Prasad S., Kumar H., Bhidayasiri R., Pal P. K. (2021). Impact of COVID-19 on patient care, training, and research in movement disorders in MDS-AOS region. *Movement Disorders*.

[71] Nandakumar S., Shahani P., Datta I., Pal R. (2021). Interventional strategies for Parkinson disease: can neural precursor cells forge a path ahead?. *ACS Chemical Neuroscience*.

